# Mutual learning and research messages: India, UK, and Europe

**DOI:** 10.4103/0019-5545.69211

**Published:** 2010-01

**Authors:** Gurvinder Kalra, Dinesh Bhugra

**Affiliations:** Senior Registrar, B.Y.L. Nair Hospital and T.N. Medical College, Mumbai - 400 008, India; 1 Professor of Mental Health and Cultural Diversity, Department of Health Service and Population Research, Institute of Psychiatry, King’s College London, De Crespigny Park, London SE5 8AF, UK

**Keywords:** Europe, India, UK, research

## Abstract

India and UK have had a long history together, since the times of the British *Raj*. Most of what Indian psychiatry is today, finds its roots in ancient Indian texts and medicine systems as much as it is influenced by the European system. Psychiatric research in India is growing. It is being influenced by research in the UK and Europe and is influencing them at the same time. In addition to the sharing of ideas and the know-how, there has also been a good amount of sharing of mental health professionals and research samples in the form of immigrants from India to the UK. The Indian mental health professionals based in UK have done a good amount of research with a focus on these Indian immigrants, giving an insight into cross-cultural aspects of some major psychiatric disorders. This article discusses the impact that research in these countries has had on each other and the contributions that have resulted from it.

## INTRODUCTION

Over six decades have passed since the British left India. However, the patterns of healthcare they had established have remained and indeed influenced the psychiatric system of modern India and continue to do so. Psychiatry has its roots as a biomedical discipline in Europe and a history related to delivery of psychiatric services largely through asylums. Certainly some facets of psychiatric services and research and training have changed as a result of changes in the healthcare policy in the last quarter of a century, although much more needs to be done. The growing research strength of Indian Psychiatry can be seen from the number of paper presentations in various conferences and the growing number of publications, some of which have been co-authored by researchers from India and UK, thus making it important to look at the impact factor that the research in both these countries have on each other. In an increasingly globalized world, not withstanding historical research, it is possible that research and service development ideas will be shared across various countries and regions. In this article we highlight some of the studies where an impact has been felt in two major regions of the world - India and Europe, including the UK.

## HISTORY

Mills[1] had divided the history of Indian psychiatry into four main periods, 1795 to 1857, 1858 to 1914, 1914 to 1947, and 1947 to the present day, and argued that the foundations of modern psychiatry in India were laid down during the British *Raj*. Parkar *et al*.[2] mentioned three major revolutions in Indian psychiatry, helping it reach where it is today. The first revolution occurred when it was believed that sin and witchcraft led to mental illness; the second revolution was the advent of psychoanalysis; and the third was the development of community psychiatry, resulting in the integration of mental healthcare in the community.

## IMPACT OF INDIAN RESEARCH ON UK

There have been several strands of research carried out in India, which have directly and indirectly influenced research and service delivery in the UK. These can be roughly divided into research, translation of research into clinical practice, and impact on service delivery for Indian patients in the UK.

Psychiatric research in India has been carried out by a number of agencies – from central organizations such as the Indian Council of Medical Research (ICMR) and Council of Scientific and Industrial Research (CSIR), to academic institutions such as National Institute of Mental Health and Neuro Sciences (NIMHANS) – Bangalore, All India Institute of Medical Sciences (AIIMS) – Delhi, and Post Graduate Institute of Medical Education and Research (PGIMER) – Chandigarh, along with NGOs such as Schizophrenia Research Foundation (SCARF), Sneha, and Sangath. Of the national organizations, ICMR is the main body in India for the formulation, coordination, and promotion of biomedical research, which also supports international collaborations, including those with the Medical Research Council, UK.[3] Mental health research is one of ICMR’s thrust research areas.

Indian psychiatry was put on the world map by the involvement of centers in Agra and Chandigarh in the two major World Health Organization (WHO) studies – International Pilot Study of Schizophrenia (IPSS) and Determinants of Outcome of Severe Mental Disorders (DOSMeD). Both these studies were the key steps in developing ideas of carrying out cross-cultural psychiatric research from the 1960s to the 1970s. Both the studies demonstrated that not only did schizophrenia exist in different parts of the world, but it had better outcomes in developing countries like India, Colombia, and Nigeria. This changed the way the world perceived schizophrenia and psychiatry as a whole in the Indian perspective. DOSMeD conducted in 1978, replicated the findings of IPSS. Speculations were made on the reasons for better outcome in developing countries, focusing on the role of families and socio-centric societies, which provided more support, placing lesser demands on the patient. These studies were the models for subsequent studies by ICMR on acute psychosis and its outcomes.

Kapur *et al*.[4] followed the development of the Present State Examination (PSE) model[5] to develop their interview schedule, the Indian Psychiatric Survey Schedule.

## ROLE OF *RAUWOLFIA*

In the 1950s, there was a revolution of sorts in the approach to psychotic disorders, with the introduction of the first pharmacological tools specifically aimed at the management of psychotic patients, with the discovery of chlorpromazine[6] and reserpine. Even as the former was a substance of chemical synthesis, the latter was an alkaloid having a natural origin, obtained from the root of *Rauwolfia serpentina*.[7] Both these drugs have helped the onset of the psychopharmacological era in the treatment of schizophrenia,[8] although the role of *Rauwolfia* is often not discussed. Commonly known as *sarpagandha* (Sanskrit name), *Rauwolfia* has been used medicinally in ancient India and has found mention in the Hindu texts dating from around 500 BC, including the *Charaka Samhita*. Vakil[9] had enumerated some 16 different names of this ‘insanity herb’. The first report on the tranquilizing effects of *Rauwolfia*, based on proper research methodology, in India, was in the 1930s,[10] although Western medicine realized its utility only after the 1940s, with the work of Indian cardiologist Rustom Jal Vakil.[11,12]

*Rauwolfia* contains an alkaloid reserpine, which reduces blood pressure,[13,14] depresses activity of the CNS[15] by depleting catecholamines and serotonin, and acts as a hypnotic.[16,17] Although the utility of *Rauwolfia* in cardiovascular disorders has been premiered by Vakil, its utility in mental illnesses can be said to have been popularized by Dr. R. A. Hakim from Ahmedabad, Gujarat, who studied the effects of ‘*Siledin*’, a mixture of medicinal plants, including *Rauwolfia*, and found it effective in schizophrenic and manic-depressive disorders.[8,18]

The early clinical trials with reserpine were carried out by Davies and Shepherd of the Maudsley Hospital, London, who found it to be useful in patients with anxiety.[19] Altschule[20] specifies that reserpine causes significant clinical improvement in psychiatric disorders such as schizophrenia, schizoaffective states, delirium tremens, catatonic syndromes, senile psychosis, and manic psychosis. Lopez-Munoz *et al*.[6] suggest that this study of Altschule might have initiated the process of deinstitutionalization of psychiatric patients. Whether the study initiated the process or not, it most certainly contributed to the likelihood that patients could be discharged and could lead better independent lives outside the asylums.

However, reserpine did not stand the test of time and was considered inferior with respect to chlorpromazine in the control of acute psychosis.[21] Despite this, other researchers like Christison *et al*.[22] and Lehman[23] have suggested the use of reserpine as one of the alternative treatments in refractory schizophrenia. Thus we see the important Indian contribution to psychiatry with *Rauwolfia*, which led to some major research influences between India and the UK.

## YOGA AND MEDITATION

Psychiatric research is also rediscovering and validating many of the ancient mental health practices from traditional cultures, and the contribution of Indian yoga in this scenario cannot be ignored. Yoga is an ancient Indian science, probably the first to recognize the interconnections between mind and body and the role this relationship plays in restoring and promoting optimal health through various postures (*asanas*), breathing exercises (*pranayamas*), and a cognitive component focusing on meditation and concentration. The *Sanskrit* meaning of yoga is ‘to unite’, which may refer to the union of mind and body through this ancient art. *Patanjali*, the foremost exponent of yoga has defined yoga as ‘*Chitta vritti nirodha*’. *Chitta* is the mind, which is constantly bombarded with ideas and thoughts, uncontrolled emotions, and worries, leading to a whirlpool (*vritti*), that needs to be controlled (*nirodh*) through *abhyasa* (constant practice) and *vairagya*, a state where feelings are not affected by environment, but by one’s own judgment.

Attempts have been made to show that yoga is an evidence-based treatment in various psychiatric disorders, from as early as 1960s, by various Indian researchers like Venkoba Rao,[24] Vahia *et al*.,[25,26] Vahia,[27–29] Udupa *et al*.,[30] Balakrishna *et al*.,[31] Sethi *et al*.,[32] Singh and Madhu,[33] Lalitha and Kaliappan,[34] Sahasi *et al*.,[35] Prabhakar *et al*.,[36] and Grover *et al*.[37] NIMHANS is also involved in yoga research for over three decades now, with ongoing research to assess the efficacy of yoga in mild cognitive impairment, Attention Deficit Hyperactivity Disorder (ADHD), and its benefit to the caregivers of schizophrenia.[38]

A 68 – 73% remission rate in the treatment of depression, regardless of the severity of depression, has been reported by the use of *Sudarshan Kriya*;[39,40] One study points to its being as effective as a standard antidepressant drug and electroconvulsive therapy (ECT),[41] and another pilot study supports its use as an adjunct treatment for depression,[42] anxiety disorders,[43,44] eating disorders,[45] alcohol dependence,[46] and even in schizophrenia.[47] Other Indian research has also shown the efficacy of yoga in post traumatic stress symptoms.[48] Recent research has highlighted the effects of yoga in chronic pain,[49] low back pain,[50,51] enhancing brain function,[52] resiliency to stress,[53,54] and the overall well-being and optimism in healthy populations,[55,56] improving sleep in the institutionalized elderly,[57] benefiting sexual drive,[58] and even smoking cessation.[59] Grover and Avasthi[60] have suggested including yoga in the Clinical Practice guidelines of the Indian Psychiatric Society.

However, of late, there has been some criticism pointing to the methodological limitations of various yoga studies.[61] The role of the patient’s attitude toward yoga is also important in predicting the response to treatment as was pointed out by Grover *et al*.[62] Increasingly in social settings in the UK, different schools of yoga are flourishing for well-being, and meditation is being used both in clinical and non-clinical services. However, not many trials have been carried out.

## SPIRITUALITY

Spirituality is distinct from religion and refers to a belief in a power greater than oneself,[63] the transcendent relationship between the person and a higher being, something that goes beyond specific religious affiliations.[64]

All around the globe there is an increased stress on a holistic approach to patients and their treatments. Indian psychiatric diaspora have led to exploring spiritual matters with their patients and the people who care for them. With the concept of holistic therapy, the therapist now has to deliver multidimensional care to the patient, with attention to the physical, mental, emotional, social, environmental, and also spiritual domains of health. Religion and spirituality are resources that help patients cope with various life stresses including that of different illnesses[65] such as arthritis[66] and cancer.[67]

Bhugra and Osbourne[68] discuss how psychiatrists are not trained to deal with religious and spiritual issues because they feel that they may be working outside the limits of their capability, pointing out how essential it is for psychiatrists to be honest and open about discussing these issues with the patients. Kumar[69] points out how Complementary and Alternative Medicine (CAM) is more popular owing to its holistic approach. To this point, Verghese[65] suggests propagating the biopsychosociospiritual model in our psychiatric approach, including religious concepts, in psychotherapy. Again, in the UK, increasing emphasis is being placed on a holistic approach of managing patients and assessment of spiritual needs is a key aspect of that.

Ravinder Lal Kapur was among the past few psychiatrists who had a special interest in spirituality, which led him to research the lives of *rishis* and *sadhus* in the Himalayas and extrapolate his findings to mental health issues.[70]

Philosophy in India is essentially spiritual, with the spiritual motive dominating Indian life. Transcendental meditation (TM), yoga therapy, and prayer are almost universally known techniques that form a part of many traditional psychotherapeutic practices still practiced in India.[71] These are a way to develop an individual’s inner self, with some people having turned to spirituality as a healing balm for their aching hearts.[72] Older literature mentions the effectiveness of meditation on anxiety states,[73] tension headaches,[74] and drug abuse.[75] Carrington and Ephron[76] have used TM as an adjunct to psychoanalysis and analytically oriented psychotherapy, and have found that it reduces tension and anxiety with improvement in psychosomatic conditions. Studies have also shown that spiritual and religious beliefs help in stress reduction,[77] pain management,[78] recovery from illness,[79] and in overcoming disability.[80,81] In a collaborative three-center study, Verghese *et al*.[82,83] have shown that those patients who spent more time in religious activities tend to have a better prognosis. Considering similar research in the UK, in a British epidemiological study by Brown and Prudo, it has been found that church going and active religion are protective of the vulnerability for depression.[84]

## UK RESEARCH ON INDIA

Of the research carried out by UK psychiatrists of Indian origin based in the UK, but with a research focus on India, Patel[85] has led the way on developing strategies of providing mental healthcare by non-psychiatrists, which includes anyone working in a healthcare setting, or who works with people who are mentally ill, but has not been trained specially for the same. These strategies have proved useful for community health workers, primary care nurses, and the social workers in developing countries. Patel[86] has also suggested new roles for psychiatrists especially from the low- and middle-income countries, in designing and managing various mental health interventions, building clinical capacity, supervision and quality assurance, and providing referral pathways and research. Patel and Bloch[87] have pointed out how the scarcity of mental health legal provisions and inadequate clinical services in the low- and middle-income countries has led to compromised mental well-being in the community.

Some of the psychiatric conditions where UK research studies have focused on India are illustrated herewith. This is in no way a comprehensive review, but it is a selective review.

## EXPRESSED EMOTION

The finding that Expressed Emotion (EE), a measure of family environment, is associated with the course of psychiatric disorder has generated a great deal of clinical and research interest in it as an important risk factor. The 1978 study of WHO, namely, DOSMeD, reported 54% households in Denmark and only 23% households in Chandigarh to be high in EE. In the latter center, urban families had a higher EE than rural families. Similar findings were reflected in the study by Leff *et al*.,[88] who found a significantly lower proportion of high-EE relatives in the Chandigarh sample and hence a better outcome as compared with a London sample.

Cheng[89] had raised important questions asking if EE could be considered an *etic* phenomenon and suggested that EE assessment needed to be recalibrated in different cultures. The sick role played by an Indian person with schizophrenia and the family’s response may have certain *emic* characteristics. It is possible that the various components of EE (Critical Comments, hostility, emotional over-involvement) in relatives of mentally ill in India may be different from their Western counterparts. In this context, Wig *et al*.[90] have found that the rating of critical comments, hostility, and positive remarks can be transferred from English to Hindi without distortion, but not the remaining two scales of over-involvement and warmth. Wig *et al*.[91] have also measured the components of EE among two samples of relatives of first-contact patients from Aarhus (Denmark) and Chandigarh (India) and found that the Danes were very similar in most respects to samples of British relatives, whereas, the Indian relatives expressed significantly fewer critical comments, fewer positive remarks, and less over-involvement. There are problems with these studies and with the approach, as no normative data were collected, and without knowing what is normal in a given population it is difficult to ascertain the true levels of EE.

## DELIBERATE SELF HARM

Examining episodes of self-harm over a one-year period in an area of London, Bhugra *et al*.[92] confirmed that women from the Indian subcontinent had the highest overall rates, especially in the younger age group, and this was attributed to social and cultural stress.[93] Subsequently Bhugra and Hicks[94] showed that the community could be educated and their knowledge of depression could be increased by using educational pamphlets on depression and suicide, especially if they were developed with the help of the community.

## EATING DISORDERS

Eating disorders like anorexia nervosa are usually seen in Euro-American societies, which lay emphasis on slimness and body image.[95] However, Bhugra *et al*.[96] hypothesized that a clash between a traditional culture and adopted culture may heighten the risk for eating and body image disturbances in susceptible individuals. This clash described by Bhugra *et al*.[96] may probably be important in the future due to the effects of globalization in India, with the Indian society being influenced by Western culture.

## COMMON MENTAL DISORDERS

According to Kirmayer and Minas,[97] globalization affects psychiatry in three main ways: through its effects on the forms of individual and collective identity, through the impact of economic inequalities on mental health, and through the shaping and dissemination of psychiatric knowledge. This latter method is important in the Indo-UK context, considering the large number of Indian psychiatrists who have migrated to the UK, either temporarily or permanently, and are now involved in sharing of the psychiatric knowledge.

The large numbers of Indians residing in the UK have provided the researchers with a special set of population sample of ethnic minority. This has encouraged research on mental health issues in them such as the study by Bhui *et al*.[98] that has found more depressive ideas in the Punjabis living in London, compared to the English general practice attendees; but they are no more likely to have somatic symptoms than the English patients.[99] In an earlier study on depression in Indian women in London, Jacob *et al*.[100] reported a low sensitivity (17%), but a high specificity (91%) in the doctor’s recognition of the condition. Similarly, Bhugra *et al*.[101] pointed out that Punjabi women, in a focus group in London, recognized the English word ‘depression’, but the older ones used terms such as ‘weight on my heart/ mind’, or ‘pressure in the mind’. The symptom of ‘sinking heart’, found in women from north India was also noted among Punjabis in the UK.[102] Studies in the UK found that South Asian immigrants, which included Indian and Pakistani individuals had lower rates of depression than the general UK population.[103] However, a later study by Bhui *et al*.[104] in the UK, using a culturally adapted measure, found higher levels of depression among Punjabi immigrants, particularly women, compared with their white counterparts in the UK. This underscores the need for culturally adapted measures, to tap expressions of distress, the so called idioms of distress that may otherwise go unreported. Bhugra and Mastrogianni[105] had enumerated some such idioms of distress.

## RESEARCH INSTRUMENTS

Universalism and relativism are the two key debates in cross-cultural psychiatric research. This debate refers to the use of either *etic* or *emic* instruments for the recognition and evaluation of mental illness. The *etic* issue of application of various diagnostic instruments on Indian patients is not a recent one, most of these being developed in the West. However, newer culturally relevant and appropriate instruments are being added, albeit at a slower pace, in the Indian research armamentarium, for example the Indian Disability Evaluation and Assessment Scale (IDEAS, 2002) developed by the Rehabilitation Committee of the Indian Psychiatric Society,[106] which has been recommended to measure psychiatric disability in four illnesses, namely, Schizophrenia, obsessive compulsive disorder (OCD), bipolar disorder, and dementia.[107] Other such instruments include the SCARF Social Functioning Index,[108] Amritsar Depressive Inventory,[109] and the like.

The Present State Examination (PSE) developed by Wing *et al*. (1974),[5] from the Social Psychiatry Research Unit of the UK Medical Research Council, is probably the most widely used instrument to record the mental state examination, and has given investigators a useful tool for reliably eliciting and recording symptoms of current psychopathology, if any. Schedules for Clinical Assessment in Neuropsychiatry (SCAN), originally called PSE, is a set of tools created by WHO, aimed at diagnosing and measuring mental illness, and can be used with either ICD-10 or DSM-IV, and includes the tenth edition of the PSE as one of its core schedules. The entire SCAN interview consists of an exhaustive 1,872 items, with most patients needing only parts of the interview.

The PSE and SCAN aim to provide comprehensive, accurate, and technically specifiable means of defining, eliciting, describing, and classifying clinical phenomena in order to make comparisons, and can be used to enhance clinical work and education and advance knowledge, through its use in epidemiological and psychosocial research.[110] These two instruments are the result of attempts to construct a simple glossary of symptom definitions and to give a structure to the way a clinical interview is to be conducted. These instruments have been extensively used in several population samples,[111] including some Indian studies,[112] and they provide an opportunity of applying uniform criteria across different centers in the world, thus making cross-cultural comparisons easy. The Hindi version of PSE has been extensively used in India, from the time of the IPSS study in the 1960s.[113]

## EUROPEAN LINKS

Learning from the experience with UK, one can say that research and training collaboration should also be encouraged and supported between the European Union and India. The relation between European and Indian psychiatry dates back to the 1850s, when Hoenigberger, a German doctor employed by *Maharaja Ranjit Singh* of Punjab, established a lunatic asylum in Patiala, Punjab. This was followed by a time when separate asylums for European mentally ill were established, and various therapies like insulin coma therapy, hydrotherapy, and various hormone extracts developed in Europe and England were industriously being followed in India. Today, there are many Indian immigrants in various countries in Europe who provide researchers with a unique ethnic sample. Migration research similar to the UK has been done in the Netherlands and the incidence of psychotic disorders was found to be high among immigrants living in neighborhoods where their own ethnic group comprised of a small proportion of the population,[114] similar to the UK studies.

Most of the psychiatric classifications originated in nineteenth century Europe as also the study of phenomenology. They were based on many philosophical concepts, which were not widely accepted in developing countries. One such example included the unconscious motivation of symptoms. This was criticized by psychiatrists from India, China, Indonesia, and the WHO, at the International Conference on the Diagnosis and Classification of Mental Disorders held in Copenhagen in 1982.[115] Today, van Os,[116] from Netherlands has proposed to introduce the diagnosis of ’Salience syndrome’ analogous to the functional-descriptive term ’Metabolic syndrome’, to replace all current diagnostic categories of psychotic disorders in DSM V and ICD 11. One has to now wait and see whether history repeats itself!

There is not much literature on the research links between Europe and India. Homeopathy developed in Germany has influenced Indian care giving in a significant way; being practiced in India for more than a century and a half now and is recognized as one of the National systems of medicine, providing healthcare to a large number of people. This system of medicine was imported into India from Germany, where Samuel Hahnemann was the major proponent of this practice. It takes a holistic approach to the patient using a single substance to treat the whole person (physical, mental, and emotional) and then allowing healing to take place. This system views all symptoms (physical, mental, and emotional) as signs of the body trying to heal.[117] In recent decades, homeopathy has been used increasingly for the treatment of medical and mental illnesses,[118] but there is little information about the outcomes from case reports or controlled studies. Similarities may exist between the basic principles of homeopathy and modern psychiatry, including the postulated role of self-healing and a possible ’microdose effect’ of treatments that permit the subsequent use of low doses to elicit therapeutic responses.[119] However, a review of 19 articles on Complementary and Alternative Medicine (CAM) by Thachil *et al*.[120] found that none of the CAM studies showed efficacy in psychiatric disorders like depression.

The Unani system of medicine also known by various names including *Al-Tibb-Al-Arabi, Al-Tibb-Al-Unani, Al-Tibb-Al-Aa’Shaab*, and so forth, owes its origin to Greece, with its theoretical framework based on the teachings of Hippocrates. It was introduced in India by Arabs. Unani physicians have all mentioned psychiatric disorders, namely, delirium, melancholia, hysteria, insomnia, and so on. They have mentioned *Quwwat-e-nafsaniya*, the psychic faculty, as one of the important faculties in the human body and have also described the causative factors, clinical features, and even the differential diagnoses of psychiatric disorders along with their management.[121] The Unani system of medicine is based on the humoral theory, which mentions four humors: Blood, Phlegm, Yellow bile, and Black bile. Every person has a unique humoral constitution, which represents the healthy state of the person with the humors in relative equilibrium. The dominance of one humor determines the *Mizaj* (temperament) of the individual, which represents the metabolic constitution, psychological makeup, and behavioral pattern of an individual. It is expressed as sanguine, phlegmatic, choleric or a melancholic temperament.[122] The dominance of phlegm or black bile determines phlegmatic or melancholic characters, respectively. Excess of humors produces diseases; for instance, black bile induces depression, while excess of yellow bile leads to hysteria and manic disorders. The aim of the Unani practitioner is to restore the normal *mizaj* of the person, through pharmacotherapy and suggestion or cognitive therapy. University pharmaceutical laboratories (such as Hamdard in Delhi and the Tibbiya College in Aligarh), as well as some private ones, offer some traditional drugs for disorders such as mania, hysteria, melancholia, sleep, and sexual disorders. However, this branch of medicine is rarely practiced today.[123] Links between Europe and India are increasing as more joint research projects are being planned and carried out. How these affect the service delivery remains to be seen.

## CONCLUSIONS

The world is changing rapidly and the challenge will be to keep pace with the latest research from the West and in the process give Indian psychiatric research a face of its own. There is also a need to raise the skills and capacity for research, by more collaborative efforts between the two countries. An even bigger challenge in doing research in India is dealing with the severely limited resources. We must not only continue to generate great ideas and knowledge, but also get better at exploiting them and exploiting ideas from elsewhere, to harvest greater benefits for the society. In order to maintain India’s position in the face of increasingly severe global competition in research, the government should adopt a clear, long-term vision for supporting the research base, and for deriving economic and social benefits from that investment.
